# Improving clinical trial efficiency by biomarker-guided patient selection

**DOI:** 10.1186/1745-6215-15-103

**Published:** 2014-04-02

**Authors:** Ruud Boessen, Hiddo J Lambers Heerspink, Dick De Zeeuw, Diederick E Grobbee, Rolf HH Groenwold, Kit CB Roes

**Affiliations:** 1 Julius Center for Health Sciences and Primary Care, University Medical Center Utrecht, Universiteitsweg 100, 3584 CG, Utrecht, The Netherlands; 2 Department of Clinical Pharmacology, University Medical Center Groningen, Antonius Deusinglaan 1, 9713 AV, Groningen, The Netherlands

**Keywords:** Clinical trial designs, Biomarkers, Baseline selection, Active run-in

## Abstract

**Background:**

In many therapeutic areas, individual patient markers have been identified that are associated with differential treatment response. These markers include both baseline characteristics, as well as short-term changes following treatment. Using such predictive markers to select subjects for inclusion in randomized clinical trials could potentially result in more targeted studies and reduce the number of subjects to recruit.

**Methods:**

This study compared three trial designs on the sample size needed to establish treatment efficacy across a range of realistic scenarios. A conventional parallel group design served as the point of reference, while the alternative designs selected subjects on either a baseline characteristic or an early improvement after a short active run-in phase. Data were generated using a model that characterized the effect of treatment on survival as a combination of a primary effect, an interaction with a baseline marker and/or an early marker improvement. A representative scenario derived from empirical data was also evaluated.

**Results:**

Simulations showed that an active run-in design could substantially reduce the number of subjects to recruit when improvement during active run-in was a reliable predictor of differential treatment response. In this case, the baseline selection design was also more efficient than the parallel group design, but less efficient than the active run-in design with an equally restricted population. For most scenarios, however, the advantage of the baseline selection design was limited.

**Conclusions:**

An active run-in design could substantially reduce the number of subjects to recruit in a randomized clinical trial. However, just as with the baseline selection design, generalizability of results may be limited and implementation could be difficult.

## Background

Clinical trials are increasingly extensive and complex [1,2]. They account for the bulk of time and money invested into drug development [3-5]. To assure the efficient and timely arrival of new and affordable drugs, it is therefore essential to explore and implement innovative approaches to the design of clinical trials [6,7].

In many therapeutic areas, prognostic research has identified subject characteristics that are predictive of future clinical outcomes or favorable/unfavorable treatment response [8,9]. These characteristics can be categorized as prognostic or predictive markers [10,11]. Prognostic markers are associated with future clinical outcomes, irrespective of treatment status, while predictive markers predict the response to treatment. Baseline albuminuria is an example of a prognostic marker that is associated with renal and cardiovascular outcomes, while unrelated to the response to angiotensin receptor blockers [12]. On the other hand, early albuminuria reduction after a relatively short duration of exposure to treatment is a predictive marker, proven to be associated with differential treatment response on long-term renal and cardiovascular endpoints in randomized clinical trials [13-15].

Information on markers associated with improved long-term treatment outcomes could be of use in improving the efficiency of clinical trials [16]. Predictive markers enable selection of patient subgroups for whom the expected effect of treatment (as compared to control) is particularly beneficial. Restricting randomization to this subgroup could reduce the sample size requirements needed to establish treatment benefit.

In this paper we distinguish selection using a baseline marker from selection on short-term changes that follow treatment. The former is applied in a baseline selection design (BSD), whereby only a selection of the recruited population (for example, those within a predefined range on the baseline marker) is randomized at baseline. Selection on short-term marker changes is applied in an active run-in design (ARD) whereby all the recruited subjects initially receive treatment, and only those with a predefined minimum improvement are subsequently selected for random allocation to treatment or control. In this case, improvement on the marker during active run-in is used as a predictive marker to guide subject selection for the randomized phase. This design has some resemblance to a randomized withdrawal design, in which subjects are all treated with the experimental treatment until response or recovery, are subsequently randomized to treatment or control and then followed for a clinical outcome (for example, a relapse). However, in the present active run-in design, the initial period is much shorter and only needed to observe a (minimum) response on a marker to guide selection.

Both the BSD and the ARD intend to increase study effect size and reduce its sample-size requirements. However, both designs do so by randomizing only part of the recruited population. Therefore, the number of subjects to recruit will be larger than the number to actually randomize. As a result, it is not evident upfront whether the BSD and ARD are more or less efficient than a conventional parallel group design (PGD) in which no selection is applied. Moreover, selection based on a predictive marker restricts the population to which the study results apply, and thus limits generalizability.

This study uses statistical simulations to compare the PGD, BSD and ARD on sample-size requirements and the generalizability of study results. A range of realistic scenarios are evaluated, including a scenario based on empirical data from two clinical trials that assessed the efficacy of antihypertensive treatments in diabetic patients.

## Methods

### Study designs

In both PGD and BSD, subjects are randomized to treatment or placebo at baseline and followed up until either the clinical endpoint or the end of the study. In PGD, the study population consists of a random sample from an unrestricted patient population. In BSD, the study population is restricted to subjects with a baseline value on a predictive marker that exceeds a predefined cutoff. Hence, only a fraction of the subjects who would have been enrolled in the PGD are actually randomized in the BSD. In ARD, all subjects start the study on active treatment and only those for whom improvement on the marker outcome after run-in exceeds a predefined minimal cutoff value are randomized to treatment or placebo in the second study stage. They are then followed up until either the clinical endpoint or the end of the study.

In both BSD and ARD, the proportion of the enrolled population that continues into the randomized study stage can be denoted by *P*, and is dependent on a selection criterion *c*. Supposing that both a lower baseline-marker level and a greater improvement on the marker during active run-in are associated with a reduced incidence rate of the endpoint in the randomized study stage. In that case, when the absolute value of c is large, *P* is small since only few subjects meet the selection criterion. Furthermore, the observed effect of treatment on the endpoint will be relatively large in the randomized population, but the fraction of the total population to which these findings apply is reduced and generalizability is limited. Conversely, when c is smaller, *p* is larger and the observed effect in the randomized population is relatively small, however generalizability improves. It is important to note that the value of *c* is a design parameter that should always be defined before the study starts to control the type-I error rate of subsequent tests. In PGD, there is no selection criterion and all recruited subjects are also randomized for follow-up.

### Simulations

A simulation study was conducted to assess the sample-size requirements of PGD (N_PGD_), BSD (N_BSD_), and ARD (N_ARD_). In all designs, subjects were randomized in a 1:1 ratio of treatment to control, either at baseline (PGD and BSD) or after an active run-in stage (ARD).

A single, large dataset (representing 100,000 subjects) was generated. Included in this dataset was the treatment status in the first (before randomization) and second (after randomization) study stage (*T*_*1*_ and *T*_*2*_, respectively with 0 representing placebo and 1 representing active treatment), and the marker level at baseline (*A*_*0*_) and after the end of the run-in stage (*A*_*1*_). Treatment status was independent of marker levels.

For PGD and BSD, the treatment status of a subject was the same in the first and second study stage (*T*_*1*_ = *T*_*2*_). For ARD, all subjects were treated in the first stage (*T*_*1*_ = 1) and randomized in the second. *A*_*0*_ and *A*_*1*_ were generated as follows: first, two series were generated from a multivariate normal distribution ~ N(3,1)) with correlation *r*. The first series represented *A*_*0*_, and *A*_*1*_ was derived as the second series minus Δ (the assumed mean improvement on the marker during the first stage). No average first-stage improvement on the marker was assumed among subjects on placebo, and so for this group Δ = 0.

Endpoint-free survival times were generated using the method described by Bender *et al.*[17]. First, a linear predictor (*lp*) was defined by:

(1) $$\mathrm{lp}=\beta_{1}T_{2}+\beta_{2}A_{0}+\beta_{3}T_{2}A_{0}+\beta_{4}T_{2}\left(A_{0}-A_{1}\right)$$

In which *β*_1_ represented the primary effect (the effect that was not mediated through the marker) of treatment in the second stage, *β*_2_ the main effect of baseline marker level (the prognostic part of the baseline marker), *β*_3_ the treatment status by baseline marker level interaction (the predictive part of the baseline marker), and *β*_4_ the treatment status by first-stage marker improvement interaction (predictive part of change on the marker). Equation 1 can be rewritten into:

(2) $$\mathrm{lp}=\left[\beta_{1}+\beta_{3}A_{0}+\beta_{4}\left(A_{0}-A_{1}\right)\right]T_{2}+\beta_{2}A_{0}$$

to show that the effect of treatment status in the second stage is a combined function of *β*_1_, *β*_3_ and *β*_4_.

Based on the linear predictor, endpoint-free survival times (*S*) were generated using:

(3) $$S=-\frac{\log\left(U\right)}{\lambda_{0}\exp\left(\mathrm{lp}\right)}$$

where *λ*_*0*_ is the baseline hazard and *U* is a random number generated from the uniform distribution *U*(0,1).

The total follow-up duration was truncated at 100 units of time for all three designs. The run-in period was set to comprise 12 percent of the total study duration (corresponding to about 3 months in a study with a 2 year total follow-up duration). Hence, while the total duration of the different designs was equal, the duration of the randomized study stage was 12 percent shorter for ARD as compared to PGD and BSD. Subjects who experienced an event during the active run-in stage in ARD were excluded from analysis, thus in principle potentially reducing the efficiency of ARD in terms of sample size as compared to the other designs. In practice this would typically result in a small number of subjects excluded, since the active run-in phase is usually only a relatively small part of the total study duration.

The performance of the three designs was evaluated across three sets of scenarios which are presented in Table 1. Within each set, multiple combinations of *β*_*3*_ and *β*_*4*_ (the predictive parts of the marker and change on the marker during run-in) were defined, and the sets differed in the values for *β*_*1*_ and *β*_*2*_ (primary treatment effect and the prognostic part of the marker). In addition, the sets differed in the value for the baseline hazard (*λ*_*0*_), which was chosen to result in equal event rates for the PGD placebo arm across the different scenarios.

**Table 1:** Parameters and parameter values that define the various simulation scenarios

**Scenario**	***r***	***Δ***	***β***_***1***_	***β***_***2***_	***β***_***3***_	***β***_***4***_
IA	0.7	0.5	0.0	0.0	0.0	−0.6
IB	0.7	0.5	0.0	0.0	−0.1	0.0
IC	0.7	0.5	0.0	0.0	−0.1	−0.6
IIA	0.7	0.5	−0.3	0.0	0.0	−0.6
IIB	0.7	0.5	−0.3	0.0	−0.1	0.0
IIC	0.7	0.5	−0.3	0.0	−0.1	−0.6
IIIA	0.7	0.5	0.0	0.5	0.0	−0.6
IIIB	0.7	0.5	0.0	0.5	−0.1	0.0
IIIC	0.7	0.5	0.0	0.5	−0.1	−0.6

*r* is the correlation between A_0_*and* A_1_. *∆* is the assumed mean improvement on the marker on treatment in the first design stage. *β*_1_ is the primary effect of treatment in the second stage, *β*_2_ is the main effect of baseline marker level, *β*_3_ is the treatment status by baseline marker level interaction and *β*_4_ is the treatment status by first-stage marker improvement interaction.

For every scenario a separate dataset was generated. From this dataset, only subjects with *A*_*0*_ > *c*_*A0*_ (subjects for whom the marker level at baseline exceeded the threshold *C*_*A0*_) were randomized in the BSD, and only those with *A*_*0*_*-A*_*1*_ > *c*_*A0-A1*_ (subjects for whom marker improvement exceeded the threshold *c*_*A0-A1*_) were randomized in the ARD. The values for *c*_*A0*_ and *c*_*A0-A1*_ were chosen to result in *P*, representing a designated percentile (100, 90, and so forth, down to 10 percent of the total population). The cutoff values were based on the entire unselected population. Obviously no subject selection was applied in the PGD (*P* = 1.0).

For every value of *P* (1.0 - 0.1), the number of subjects to include to significantly establish treatment efficacy in the corresponding stratum was estimated based on a log-rank test with 80 percent statistical power and a nominal type-I error rate of 5 percent, two-sided. The process of generating a dataset and estimating the required sample size for the various strata was repeated 100 times, and estimates were averaged across replications in order to reduce random simulation error. The resulting value represented the number of subjects to be randomized, and was multiplied by the inflation factor 1/*P* in order to obtain the number of subjects to be recruited. For the PGD the same steps were performed, but since no subject selection was applied, the number of subjects to be randomized equaled the number to be recruited.

The data simulated for subjects in a selected stratum could be used to derive an (extrapolated) overall effect-size estimate (hazard ratio) for the overall unrestricted population based on the regression model, with parameters estimated from the data in that particular replication. This reflects the situation whereby a particular selection design is carried out and a treatment effect for the total population is estimated from the results of that specific trial. Obviously, this overall effect-size estimate becomes less precise when it is derived from an increasingly restricted subgroup. We evaluated the precision of these estimates for the various strata in both ARD and BSD by estimating the effect-size for the overall population from the various strata and replicating this process 1000 times.

### Empirical example of antihypertensive trials with diabetic patients

We evaluated three designs for a scenario derived from the data of two empirical studies. The Reduction in End Points in NIDDM (noninsulin-dependent diabetes mellitus) with the Angiotensin II Antagonist Losartan (RENAAL) study and the Irbesartan Diabetic Nephropathy Trial (IDNT) were both multinational, randomized, double-blind trials with a renal endpoint (development of end-stage renal disease or death from any cause), conducted in patients in advanced stages of diabetic nephropathy [18,19]. The RENAAL and IDNT trials involved 1513 and 1715 patients respectively. In the RENAAL trial, patients received losartan (either 50 or 100 mg/day) or placebo. In the IDNT trial, patients received irbesartan (300 mg/day), amlodipine (10 mg/day) or a matched placebo. Both trials were designed to compare an angiotensin receptor blocker based antihypertensive regimen with a conventional blood pressure lowering regimen. To this end, blood pressure was targeted to achieve a blood pressure goal of less than 140/90 mmHg. If the blood pressure target was not achieved, additional antihypertensive agents (but not ACE-inhibitors or angiotensin receptor blockers) were allowed throughout the study. The average follow-up time was 3.4 years for the RENAAL study and 2.6 years for IDNT. Several clinical and laboratory characteristics were assessed at regular intervals during the trials. These included a measurement of albuminuria at baseline and after 3 months of follow-up. For the present study, the data from the losartan and irbesartan arms were pooled into a single active-treatment group and compared to the pooled data from both placebo arms. For the purpose of analysis, the amlodipine arm (567 subjects) of the IDNT trial was excluded. The distribution of albuminuria levels was first normalized by applying a log-transformation, and then standardized and shifted three units upward, in order to allow for comparisons with the results from our simulations. Follow-up was truncated at 750 days (approximately 2 years), again to allow for comparisons.

The data were fitted to the model represented by Equation 1, in order to estimate the effects of treatment, biomarker levels, and their interactions on the outcome. The other parameters used in the simulations (*r*, Δ, and *λ*_0_) were also derived from the empirical data. The resulting scenario was evaluated using the simulation approach described above. This allowed us to determine the number of patients to recruit for the PGD and for the various strata in the ARD and BSD for this particular empirical example.

## Results

**Figure 1:**
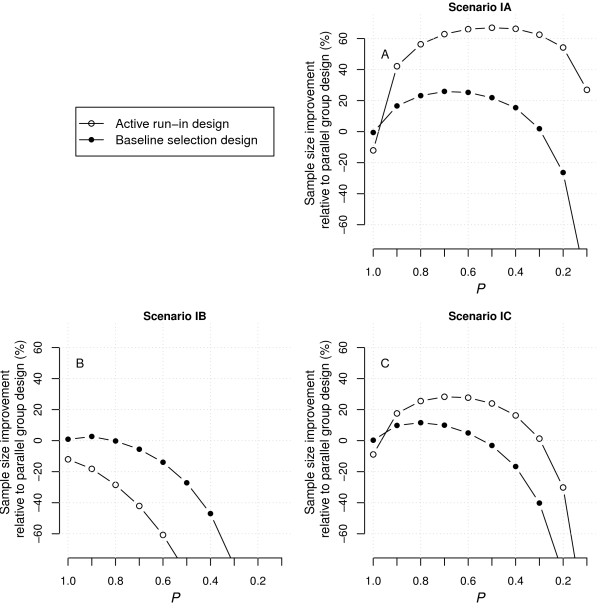
**Improvement rates in the number of patients to recruit for ARD and BSD as compared to PGD under scenarios IA-C.** Positive improvement indicates smaller sample sizes and thus higher efficiency. The sample size requirements for the parallel group design were 1386, 800 and 318 for scenario IA-C, respectively. ARD, active run-in design; BSD, baseline selection design; PGD, parallel group design.

Simulation results for the first set of scenarios are presented in Figure 1. These scenarios did not include a primary effect of treatment unrelated to baseline marker level or short-term marker improvement (*β*_1_ = 0), nor an association between baseline marker level and outcome incidence (*β*_2_ = 0). In other words, baseline marker level was not included as a prognostic factor for survival, and the full effect of treatment as compared to placebo could be predicted from baseline marker levels or early marker improvements during the run-in stage.

In scenario IA, the effect of treatment was fully expressed as part of the interaction with early marker improvement (*β*_3_ = 0, *β*_4_ ≠ 0). In this case, both ARD and BSD had the potential to reduce sample-size requirements in comparison to PGD. For ARD in particular, the increase in treatment effect from the unselected population to more restricted patient strata outweighed the loss of efficiency due to the exclusion of subjects that did not meet the selection criteria. With optimal restriction (*P* = 0.5), ARD required a little under one third of the sample size that was required with PGD. Further restriction reduced the comparative efficiency of ARD, since the further increase in treatment effect no longer outweighed the exclusion of subjects after the run-in stage. It should be noted that when *P* = 1, ARD required slightly more subjects than either PGD or BSD (a general picture seen in all evaluated scenarios), as events during the active run-in stage were excluded in the analysis, leading to a slightly smaller total event rate in ARD. BSD was also more efficient than PGD as the higher baseline marker levels were correlated with larger marker improvement and thus a larger effect on survival. As a result, subjects in the more restricted strata generally displayed a larger marker improvement and hence a stronger effect of treatment. At the optimal level of restriction (*P* = 0.7), BSD was about 30 percent more efficient than PGD, but still less efficient when compared to ARD.

When the effect of treatment was fully expressed as part of the interaction with baseline marker levels (*β*_3_ ≠ 0, *β*_4_ = 0; scenario IB), neither BSD nor ARD were more efficient than PGD for any value of *P*. In this case, the larger treatment effect in more restricted strata was cancelled out by the increasing proportion of subjects that were excluded after selection. ARD was less efficient than BSD as improvement on the marker during run-in was only partly related to baseline marker level, and hence to an increase in effect size with more restricted strata. In general, subjects with a larger short-term improvement had a higher baseline marker level to start with, and while improvement in the marker in itself was unrelated to survival in this scenario (*β*_4_ = 0), baseline marker level was not (*β*_3_ ≠ 0).

When the treatment effect could be predicted by the baseline marker level as well as short-term marker improvement (*β*_3_ ≠ 0, *β*_4_ ≠ 0; scenario IC), ARD and BSD had some potential to increase efficiency when compared to PGD, but considerably less than when the treatment effect was only included as part of the interaction with marker improvement (scenario IA). For ARD, the increased treatment effect in more restrictive strata still outweighed the exclusion of subjects after run-in, but only up to a certain degree of restriction. Further restriction (*P* < 0.2) dramatically reduced its efficiency relative to PGD. The largest increase in efficiency of ARD corresponded to approximately 25 percent fewer subjects than PGD. For BSD, the maximum efficiency gain was only 10 percent.

**Figure 2:**
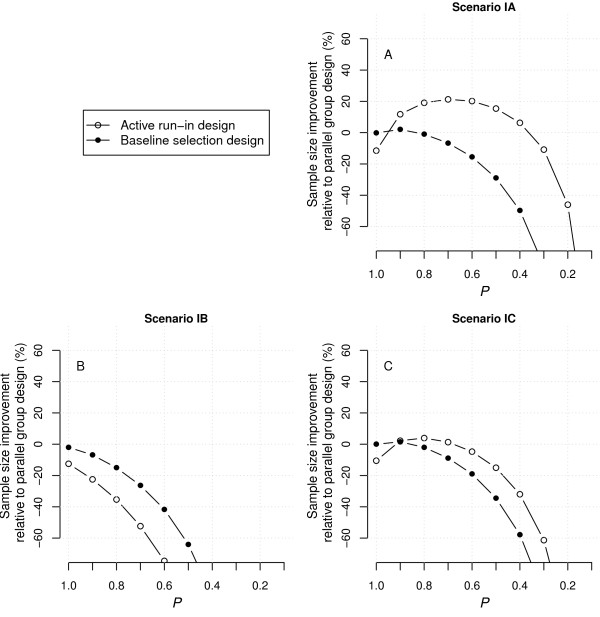
**Improvement in the number of patients to recruit for ARD and BSD as compared to PGD under scenarios IIA-C.** Positive improvement indicates smaller sample sizes and thus higher efficiency. The sample size requirements for PGD were 298, 228 and 146 for scenario IIA-C, respectively. ARD, active run-in design; BSD, baseline selection design; PGD, parallel group design.

Figure 2 shows the results for the second set of scenarios which all included a primary effect of treatment (*β*_1_ ≠ 0), which meant that part of the long-term treatment effect was unrelated to baseline marker level or short-term marker improvement. These scenarios did not include an association between baseline marker level and outcome (*β*_2_ = 0). In this case, sample sizes were reduced overall since the total effect of treatment was a combined function of *β*_1_, *β*_3_ and *β*_4_ (Equation 2), and therefore larger than in the scenarios of the first set. In general, the results showed the same patterns as observed for the first set, but the potential for an efficiency gain (for ARD and BSD as compared to PGD) was reduced over the whole range of *P*. This resulted from the fact that the relative difference in treatment effect associated with an increase in *β*_3_ and/or *β*_4_ was smaller.

**Figure 3:**
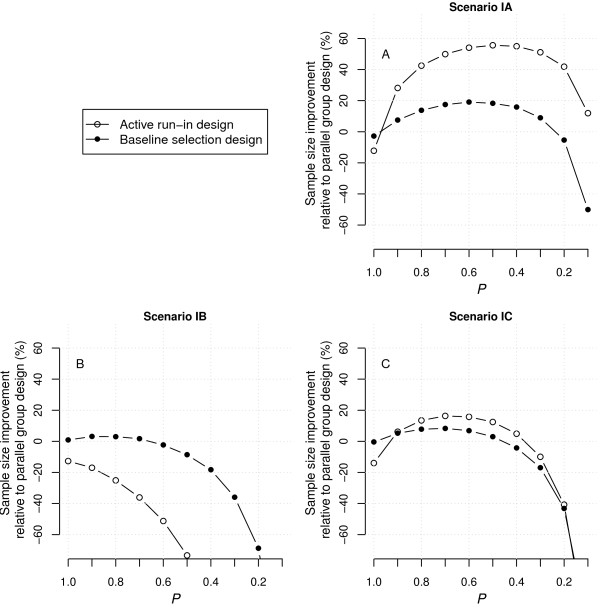
**Improvement in the number of patients to recruit for ARD and BSD as compared to PGD under scenarios IIIA-C.** Positive improvement indicates smaller sample sizes and thus higher efficiency. The sample size requirements for the PGD were 900, 692 and 236 for scenario IIIA-C, respectively. ARD, active run-in design; BSD, baseline selection design; PGD, parallel group design.

Figure 3 presents the results for the third set of scenarios, which are very similar to those for the first set. Scenarios IIIA-C included an association between baseline marker level and outcomes (*β*_2_ ≠ 0), but no primary effect of treatment independent of the interaction with baseline marker level or short-term marker improvement (*β*_1_ = 0). Equation 2 already demonstrates that *β*_2_ had no influence on the treatment effect size, but instead resulted in an overall increase in event rate (for both the control and the treatment group), which was corrected for by a lowering of the baseline hazard.

**Figure 4:**
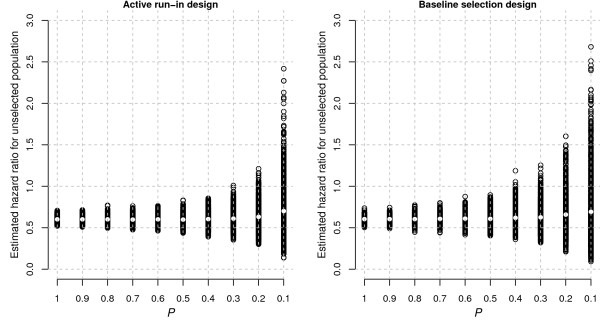
**Estimates of the hazard ratio for the overall (unrestricted) population as derived from the various patient strata in ARD and BSD.** Each circle represents the estimate from a single replication. The white dot is the average estimate over 1000 replications. ARD, active run-in design; BSD, baseline selection design.

Figure 4 shows the estimates of the effect size in the full (unselected) patient population as derived from the various patient strata in ARD and BSD. As expected, the imprecision of these effect-size estimates increased when they were derived from increasingly restricted, and thus smaller strata. In other words, there is an increased risk that the extrapolated effect-size estimate for the unselected population is biased when it is derived from increasingly restricted strata. The imprecision is larger for BSD than for ARD, because the time between patient selection and end of follow-up was shorter in ARD.

**Figure 5:**
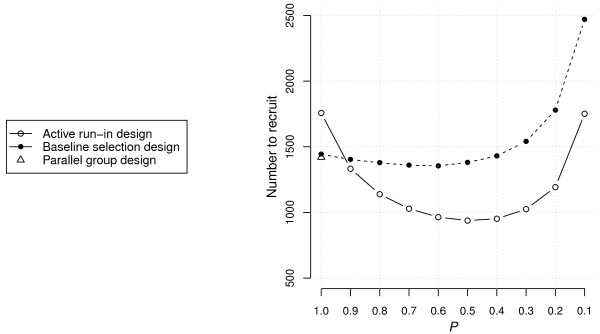
**Sample-size requirements for ARD, BSD and PGD under the scenario based on empirical data from two clinical trials that assessed the efficacy of antihypertensive treatments in diabetic patients.** ARD, active run-in design; BSD, baseline selection design; PGD, parallel group design.

Finally, Figure 5 shows the results for the scenario that was derived from empirical data. This scenario was characterized by the following parameters: *β*_1_ = -0.25 (*P* = 0.51), *β*_2_ = 1.10 (*P* < 0.01), *β*_3_ = 0.05 (*P* = 0.61), *β*_4_ = -0.49 (*P* < 0.01), *r* = 0.79, Δ = 0.32, and *λ*_0_ = 7.7e^−5^. These results indicate that baseline albuminuria is a strong prognostic factor for endpoint-free survival, but not significantly associated with differential treatment response, while early improvement in albuminuria is significantly associated with differential response to treatment. In addition, the primary effect of treatment was small and not significant. Clearly, these estimates were derived from a model that may not have been the most adequate representation of these data. As a result, they should be interpreted with care.

The simulation results indicate that BSD does not have large potential to increase efficiency when compared to PGD. In contrast, ARD did have the potential to increase efficiency. With an unselected population (*P* = 1.0) the ARD required about 20 percent more subjects than the PGD, but with optimal restriction (*P* = 0.5) the advantage was about 35 percent (Figure 5).

## Discussion

This study evaluated the sample-size requirements (the number of subjects to be recruited) to establish treatment efficacy for three study designs across a range of realistic scenarios. These scenarios characterized the effect of treatment on endpoint-free survival as a combination of an interaction with a baseline marker and/or short-term marker improvements in addition to a primary effect that was unrelated to both. The designs include: a conventional PGD with an unselected study population, a BSD that is similar to the PGD, but randomizes only a selection of the recruited population based on a predictive baseline marker, and 3) an ARD that exposes all subjects to a short treatment run-in phase and randomizes only those with a predefined improvement on the marker during the run-in stage.

Findings indicated that ARD is generally the most efficient design to establish treatment efficacy when short-term improvements during the active run-in stage are a reliable predictor for the long-term effect of treatment on survival. In the presence of a reliable baseline predictor or a primary treatment effect (unrelated to any observed marker value) the advantage of ARD decreased.

When short-term improvements were strongly related to long-term survival, BSD had an advantage over PGD as higher baseline marker levels were correlated with larger short-term improvements. When only the baseline marker level in itself was predictive of treatment efficacy, the observed effect in BSD was larger than in PGD, but did not result in a more efficient design as a portion of the recruited population was excluded in the selection. In none of these cases was there any meaningful advantage of baseline selection.

An additional scenario was evaluated that was based on empirical data from two diabetes trials. Here, baseline albuminuria was a prognostic biomarker for endpoint-free survival, and short-term reduction in albuminuria after treatment was predictive of differential treatment response on survival. In this scenario, ARD had the potential to reduce sample-size requirements as compared to PGD with up to 35 percent. This efficiency advantage would have been achieved by randomizing only the top 50 percent of the recruited population with the largest early reduction in albuminuria during the run-in phase.

While in this example patient selection was based on a predictive biomarker, it could also be based on a genomic marker [20] or a risk score from a prognostic model [21]. The use of genomic-based predictive biomarkers is an area of great research interest in oncology. The discovery of genes that have been proven to be of clinical relevance such as the *Her2/neu* gene in breast cancer and epidermal growth factor 1 (*EGFR1*) in non-small cell lung cancer has intensified interest in this area. Mutations in these genes can be used for baseline patient selection who are more susceptible for intervention. It has been shown that *Her2/neu* positive tumors are more aggressive. Patients with *Her2/neu* overexpressing tumors benefit from trastuzumab, a monoclonal antibody against the *Her2/neu* receptor. It should be noted that not all patients with positive *Her2/neu* status respond to trastuzumab, which may be due to primary or acquired resistance against trastuzumab [22,23]. With regards to *EGFR1*, two small molecule tyrosine kinase inhibitors (gefitinib and erlotinib) against *EGFR1* are available. Emerging data demonstrate that among non-small-cell lung carcinoma patients mutations in the tyrosine kinase domain (exons 18, 19 and 21) are predictive of the response to gefitinib and erlotinib [24,25]. Mutations in the tyrosine kinsase domain stimulate *EGFR1* signaling, thereby increasing susceptibility to *EGFR1* inhibitors and amplifying clinical responsiveness. More examples about genomic predictive markers in oncology are reviewed elsewhere [26]. With respect to enrichment design, a recent study has suggested that among lung cancer patients, selecting responder patients after two cycles of chemotherapy in combination with histological scoring may predict long-term survival benefit [27]. Further studies are required to confirm these findings. An example of predictive short-term change with treatment includes the lowering of LDL cholesterol and the effect of lipid-modifying therapies on the risk for cardiovascular events [28].

Both BSD and ARD apply selection based on an individual patient measure that is associated with differential treatment response. This measure is obtained either at baseline (BSD) or after a relatively short exposure to treatment in an active run-in stage (ARD). In statistical terms, there is an interaction between this measure and the long-term effect of treatment on the outcome. In biological terms, there may be an undisclosed underlying mechanism to explain the interaction. As a result, every conceivable gain in efficiency (with BSD or ARD) is associated with a reduction in the generalizability of the trial results. This results from the fact that the selected and randomized subgroup is unrepresentative of the entire patient population. Moreover, it becomes increasingly difficult to extrapolate the observed effect to the overall population when selection becomes increasingly restrictive.

As a result, findings from BSD and ARD apply primarily to the selected patient subgroup and cannot be readily extrapolated to a broader population without running the risk of introducing bias. This complicates general statements on the efficacy of the investigated drug and illustrates an important concern regarding the use of these designs in confirmatory clinical trials. In a sense, both BSD and ARD generate a substantial amount of “missing data”, which is to say, information on the (long-term) efficacy and safety of the drug that will be missing for the deselected population. In this respect, ARD has an advantage over BSD in that it collects considerable information on the short-term effects of the experimental treatment during the run-in phase. Both ARD and BSD are likely most beneficial in situations where a meaningful effect of treatment in the total population is unlikely, but valid markers are available to reliably identify patient subgroups where benefit from treatment is improved.

There is also a difference in the clinical implications of the results from both designs. BSD reflects a situation whereby prescription of the drug is restricted to patients with a specific baseline characteristic that is related to its outcome, which is something that often occurs in clinical practice as well. ARD, on the other hand, reflects a situation where the drug is initially prescribed to everyone and early improvement on a measurable marker decides whether treatment is continued or aborted.

The practicality of BSD and ARD is obviously dependent on the availability of a valid and reliable predictive marker that can identify a patient subgroup most likely to benefit from treatment. Appropriate markers are those on the causal path from treatment to effect and are preferably identified in earlier (pre-clinical) studies. If long-term efficacy of the investigated treatment is unrelated to an available marker, the enrollment of only marker-positive subjects may slow down recruitment, increase expenses, and unnecessarily limit the size of the indicated population. As to be expected, both ARD and BSD were also found to be less efficient than PGD in this scenario (results not shown). If the investigated treatment truly benefits a specific subgroup but the marker used for selection is unfit to accurately identify that group, a beneficial treatment could mistakenly be abandoned. It is however, notoriously difficult to identify genuine predictors of differential treatment response. Such evidence is usually accumulated slowly from secondary analyses of existing trials and meta-analyses.

In the current study, data were generated using a fairly straightforward model that divided the total effect of treatment on long-term survival into three components (a primary effect, an interaction with a baseline marker and an interaction with short-term improvement on treatment), and in addition included the baseline marker as a prognostic marker for survival (unrelated to treatment status).

When the effect of treatment was fully accounted for by its interaction with the baseline marker, the increase in efficiency that resulted from larger effect sizes in more restricted strata was cancelled out by the exclusion of recruited subjects after selection. In this case, both BSD and ARD had very limited potential to increase efficiency as compared to PGD. This resulted from the fact that baseline marker levels were linearly associated with the linear predictor (Equation 1) and exponentially associated with the treatment vs. control hazard ratio (Equation 3). Consequently, the additional reduction in the number to randomize decreased exponentially with further restriction of the indicated population. When restriction was more extreme, the exclusion of subjects after selection started to outweigh the advantage of larger effects in the more restricted strata. Under these circumstances, BSD was only more efficient than PGD when there was a very strong (and unrealistic) interaction effect between baseline marker level and treatment status, which was not included in the evaluated scenarios.

Some consideration regarding our model should be mentioned. An increase in the prognostic effect of baseline marker level increased the treatment effect size, and hence reduced sample-size requirements despite adjustment of the baseline hazard, to result in equal event rates for the control group as observed in the other scenarios. When data were simulated based on a model that included a prognostic effect of the baseline marker on survival there was more variation in the individual hazards. This caused some subjects to develop the endpoint early in the trial, irrespective of their treatment status, whereas others were very unlikely to experience the endpoint during the trial. The net effect was that the probability of the outcome after a certain period of follow-up was lower among treated subjects compared to the treated subjects in the simulations based on the model without the prognostic effect of baseline marker level on survival.

The model used is fairly flexible and fitted the experimental data well. However, results and conclusions may not hold to the same extent if a substantially different model is used. This will particularly be the case if the underlying proportional hazards assumption does not apply.

It should also be noted that the improvement in efficiency with BSD and ARD as reported in this study is relative to a PGD that disregards baseline marker level and early changes in the marker in response to treatment as a covariate in the analysis of the data. Including these factors as covariates in the final drug efficacy analysis would improve the efficiency of the PGD, and hence reduce the comparative advantage of both ARD and BSD.

## Conclusions

In summary, our results suggest that an ARD can substantially reduce the number of subjects to recruit in a clinical trial when short-term improvement on the marker during the run-in phase is a strong and reliable predictor of differential treatment response. Under these conditions, BSD was also potentially more efficient than PGD, but always less efficient than ARD given equally restricted strata. For all other scenarios evaluated, no meaningful advantage was observed for BSD. Generalizability issues may limit the applicability of ARD and BSD in practice. In addition, valid markers must be available to reliably identify patient subgroups with an increased likelihood to eventually benefit from investigational treatment.

## Abbreviations

ARD: active run-in design; BSD: baseline selection design; PGD: parallel group design.

## Competing interests

The authors declare that they have no competing interests.

## Authors’ contributions

RB contributed to the conception and design of the study, data collection and analysis, manuscript writing and final approval of the manuscript. HJLH contributed to the conception and design of the study, manuscript writing and final approval of the manuscript. RHHG contributed to the conception and design of the study, manuscript writing and final approval of the manuscript. KCBR contributed to the conception and design of the study, manuscript writing and final approval of the manuscript. DZ contributed to the conception and design of the study, manuscript writing and final approval of the manuscript. DEG contributed to the conception and design of the study, manuscript writing and final approval of the manuscript. All authors read and approved the final manuscript.
